# Editorial: Chemokines and Bone

**DOI:** 10.3389/fendo.2018.00386

**Published:** 2018-07-19

**Authors:** Annette Gilchrist, Paula H. Stern

**Affiliations:** ^1^Department of Pharmaceutical Sciences, Midwestern University, Downers Grove, IL, United States; ^2^Department of Pharmacology, Feinberg School of Medicine, Northwestern University, Chicago, IL, United States

**Keywords:** cytokine, chemokine, osteoclast, osteoblast, stromal cell

According to Webster's dictionary, equilibrium is a state of balance between opposing forces or actions. Bone provides a great example of why a dynamic equilibrium is important. The balance between bone resorption and bone formation is critical for the maintenance of bone mass and systemic mineral homeostasis. If there is excessive resorption by osteoclasts without a corresponding response by osteoblasts the result is osteoporosis, whereas the contrary may result in osteopetrosis. This equilibrium is dependent on a number of competing and complementary influences such as hormones, growth factors, biomechanical stimulation, and chemokines. Chemokines are a type of cytokine, defined by their ability to induce directional cell migration. Cytokines are a diverse group of soluble peptides and proteins including interleukins, interferons, colony stimulating factors, and chemokines that act as humoral regulators at nano- to pico-molar concentrations. The articles assembled for this Frontiers Research Topic focus on the chemokines and how they influence bone. In addition to four reviews covering a range of topics [orthopedic implant debris (Hallab and Jacobst), post-fracture inflammation and healing (Edderkaoui), their role in metastatic bone tropism (Coniglio), and their influence on PTH on bone remodeling (Siddiqui and Partridge)] there is a hypothesis and theory article discussing environmental factors that impact bone-relevant chemokines (Smith et al.) and an original research article on how differentiation of preosteoblast cells to adipocytes is influenced by 1,25(OH)_2_ Vitamin D_3_ (Pendleton and Chandar).

Drs. Nadim Hallab and Joshua Jacobs provide a review on the chemokines associated with pathologic responses to orthopedic implant debris (Hallab and Jacobs). While hip and knee replacements have become commonplace in part due to their success, the rate of failure grows each year following surgery due to a progressive inflammatory state that compromises the bone implant surface. The review covers activation of the innate and adaptive immune systems by implant debris (e.g., wear particles and metal ions), and how these events subsequently lead to loosening, a serious complication. They point out that aseptic implant failure due to inflammation is responsible for over 70% of total hip arthroplasty revisions and over 44% of total knee arthroplasty revisions (1). The migration of macrophages and osteoclasts to the implant site via chemokine-induced inflammatory responses leads to accelerated osteolysis and implant failure. The chemokines central to this (IL-8, MCP-1, MIP-1α/MIP-1β, CCL17, and CCL22) as well as their target cells (macrophage, lymphocytes, osteoclasts, osteoblasts, fibroblasts) are discussed. Their conclusion, that inhibiting a single chemokine is unlikely to succeed clinically given the pleiotropic nature of chemokines and their receptors, is one that is repeated by other articles provided in this Frontiers Research Topic.

Dr. Bouchra Edderkaoui reviewed chemokine expression in response to bone fracture and the potential role of chemokines in post-fracture inflammation and healing (Edderkaoui). As delayed and non-union bone fracture healing are important clinical problems, the chemokines thought to play an important role in each of the three phases of fracture healing (inflammation, bone formation, bone remodeling) are discussed. In addition, based on chemokine expression patterns and animal models utilizing knockout mice lacking select chemokines or their receptors, a hypothetical model for the contributions of chemokines in fracture healing is presented. While the importance of some chemokines seems clear (e.g., CCL2 and CXCL12), other chemokines (CXCL8, CCL4, CCL7) may also be involved in fracture healing.

Drs. Jawed Siddiqui and Nicola Partridge provide a review on the pharmacological role of Parathyroid Hormone (PTH) and the involvement of CCL2/Monocyte Chemoattractant Protein 1 in the process of PTH-mediated bone remodeling (Siddiqui and Partridge). The contribution of CCL2 and its receptor CCR2 have been established for a number of pathological conditions that involve bone including rheumatoid arthritis, cancer-induced bone loss, and bacterially induced bone loss. The researchers provide the rationale for focusing on CCL2 and its receptor CCR2 given their prominence in bone remodeling and role in osteoclast recruitment, differentiation, and fusion. They point out their work establishing the robust increase in CCL2 following intermittent PTH(1-34) injections (2). CCL2 null mice are unable to increase bone mineral density and bone volume (2), and are protected against PTH-induced cortical and trabecular bone loss (3). Thus, they suggest CCL2 expression by osteoblasts mediates the anabolic effects of PTH on bone, specifically that transient upregulation (vs. sustained) of CCL2 plays an important role in the bone resorption that ultimately increases net bone formation.

That many tumors exhibit tropism with respect to metastasis has been recognized for over a century. Stephen Paget's “seed and soil” hypothesis (4) proposing that organ-preference patterns of tumor metastasis result from favorable interactions between metastatic tumor cells (the “seed”) and their organ microenvironment (the “soil”) has been validated numerous times over. Dr. Salvatore Coniglio reviews the role of chemokines, focusing on CCL2, CCL3, IL-8/CXCL8, and CXCL12, in promoting osteoclastogenesis and tumor growth and evaluates the use of neutralizing antibodies and chemokine receptor specific antagonists as a means of chemotherapy (Coniglio).

A number of chemokines impact bone, having diverse effects on chondrogenesis, osteoblast differentiation, mineral opposition, fracture healing, osteoclastogenesis, and osteoclast activity, playing a role in the success and failure of implants, the bone loss in rheumatoid arthritis and the cancer-related bone loss and metastases (5). The comprehensive review by Smith et al. reveals an extensive literature on environmental factors impacting bone-relevant chemokines and points out many potentially fruitful areas of research into the role of specific factors either as agents interfering with normal bone development and healing, or alternatively as potential bone therapeutic agents.

Another agent with marked effects on both bone and chemokines is vitamin D. Vitamin D was first identified as an anti-rachitic factor, shown to function by increasing calcium absorption from the intestine and thereby enhancing bone mineralization. In the 1980's, high affinity binding sites for the active metabolite, 1,25-(OH)_2_ D_3_ (calcitriol) were found to be widely expressed, including in cells of the immune system (6). Calcitriol was observed to inhibit proliferation of stimulated peripheral blood mononuclear cells (7, 8) and promote their differentiation (9). The establishment of the role of locally produced cytokines and chemokines in mediating tissue effects of inflammation led to investigation of the effects of vitamin D metabolites and analogs on this and other chemokine actions. Examples of interactions in the immune system are effects of the vitamin D analogs BXL-01-0029 and elocalcitol to suppress release of CXCL10, a chemokine driving Th1-mediated inflammation (10), and the actions of calcitriol on the maturation and migration of dendritic cells, with inhibition of production of IL-1α and impairment of chemotactic responses to CCL4 and CCL19 (11). Among relevant actions in other tissues, elocalcitol inhibited tumor necrosis factor-induced CXCL10 secretion in human thyrocytes (12), the analog BXL-219 reduced production of CXCL10, CCL2 and CCL5 in mouse pancreatic islet cells (13), and calcitriol decreased CCL2, CCL5 and CXCL8 secretion elicited by macrophage conditioned medium in human adipocytes (14). In periodontal ligament cells, a connective tissue in which calcitriol increases osteogenic markers (15), calcitriol inhibited the lipopolysaccharide-stimulated production of CCL1 (15) and CCL2, CXCL8 (16). A study in osteoclasts revealed that calcitriol inhibited basal and IL-1α-stimulated production of CXCL8 (17). Interestingly, Pendleton and Chandar found that exposure of preosteoblast MC3T3-E1 cells to media utilized to promote adipogenesis could be influenced by calcitriol. The wide range of effects of chemokines on bone (5) and the marked effects of vitamin D compounds in chemokine production make the involvement of chemokines in other actions of vitamin D compounds on bone an important topic meriting further investigation.

## Author contributions

All authors listed have made a substantial, direct and intellectual contribution to the work, and approved it for publication.

### Conflict of interest statement

The authors declare that the research was conducted in the absence of any commercial or financial relationships that could be construed as a potential conflict of interest.
